# Soil phosphorus forms and their availability in six typical plantations at the southern foot of the Taihang Mountains, China

**DOI:** 10.1038/s41598-026-45512-2

**Published:** 2026-03-26

**Authors:** Jingjing Zhuang, Yun Ma, Cui Cheng

**Affiliations:** 1 School of Civil Engineering and Architecture, Xinxiang University, Xinxiang, 453003 Henan China; 2 Hebi Academy of Agricultural Sciences, Hebi, 458030 Henan China

**Keywords:** Plantation forest, Forest soil, Phosphorus fractionation, Soil fertility, Mixed forest, Environmental monitoring, Ecology, Ecology, Environmental sciences

## Abstract

Assessing the differences in soil phosphorus (P) components between pure and mixed plantations is crucial for understanding the mechanisms of P retention and release in forest ecosystems. This study aimed to evaluate the forms and availability of soil P across different plantation types to inform sustainable forest management practices.We employed a modified Hedley phosphorus fractionation scheme to analyze soils from six plantation stands: pure forests of *Robinia pseudoacacia* (*R*), *Platycladus orientalis* (*P*), and *Quercus variabilis* (*Q*), and their mixed combinations (*RQ*, *RP*, *PQ*). The results demonstrated that: (1) Organic P was the dominant form across all forest types, with significantly higher levels in mixed forests than in pure forests. Both inorganic and organic P decreased with increasing soil depth, with the highest total P concentrations found in the 0–10 cm layer of the RQ forest. Notably, the maximum occluded P (OP) content (193.23 mg·kg⁻¹) was recorded in the 20–30 cm layer of the RQ forest. (2) Phosphorus fractions followed the order: occluded P (OP) > moderately active P (MAP) > readily reactive P (RRP) ≈ available P (AP). (3) Inorganic and organic P showed significant positive correlations with OM, TC, TN, TK, TP, and TOC (*P* < 0.01). AP, RRP, and MAP were also positively correlated with these soil properties, whereas OP was negatively correlated. Our findings suggest that mixed forests, particularly the *R. pseudoacacia*–*Q. variabilis* combination, were associated with enhanced soil P retention and availability. Therefore, forest management strategies in this region should consider mixed-species plantations to optimize P utilization efficiency. These findings provide a scientific basis for improving soil fertility and sustainable P management in afforestation projects.

## Introduction

Phosphorus (P) is an indispensable element for plant growth, development, and metabolic processes, serving as a key limiting nutrient in many terrestrial ecosystems and playing a critical role in maintaining ecosystem structure and function^1^. The primary source of P for plants is the soil, where its bioavailability is governed by a complex interplay of chemical, physical, and biological processes that transform P among various inorganic and organic forms^2^. Understanding the dynamics of these soil P forms—their pool sizes, transformations, and vertical distribution—is therefore fundamental for assessing soil fertility, ecosystem productivity, and nutrient management strategies in forest ecosystems^3^.

The Hedley sequential phosphorus fractionation method, and its subsequent modifications, have become a pivotal tool for characterizing soil P status beyond simple available P measurements^4^. This approach operationally defines P pools ranging from readily available to recalcitrant, providing insights into the lability and potential turnover of soil P^5^. Applying such fractionation schemes is particularly relevant in the context of global environmental changes, which can alter P cycling pathways and necessitate a nuanced understanding of P dynamics^6^.

In North China, large-scale afforestation has been implemented for soil and water conservation, with species such as *Robinia pseudoacacia*, *Platycladus orientalis*, and *Quercus variabilis* being widely planted. However, long-term monocultures, especially of *R. pseudoacacia*, have often been associated with soil degradation, including declines in soil organic matter and available P^7^. In contrast, mixed-species plantations are increasingly advocated for their potential to enhance ecosystem multifunctionality and nutrient cycling^8^. Yet, a critical knowledge gap persists: the benefits of species mixing are unlikely to be uniform. The magnitude and mechanisms of such benefits are hypothesized to depend fundamentally on the functional identity of the component species and the nature of their interactions^8^.

The three species in this study represent distinct belowground ecological strategies: *Robinia pseudoacacia* is a nitrogen-fixing legume capable of forming symbiotic associations with both rhizobia and arbuscular mycorrhizal (AM) fungi; *Quercus variabilis* is predominantly associated with ectomycorrhizal (ECM) fungi, a guild known for their proficiency in mobilizing organic P through enzymatic activity; and *Platycladus orientalis*, also an ECM-associated conifer, may differ in root architecture and carbon allocation patterns^9^. These divergent strategies—N-fixation, AM, and ECM—set the stage for potential functional complementarity or redundancy when species are grown in mixture. For instance, the N-rich litter and root exudates from *Robinia* could stimulate microbial activity and P mineralization, while ECM fungi from *Quercus* could enhance access to different P pools. However, empirical evidence linking such specific trait combinations to soil P form differentiation in temperate plantations remains scarce.

Furthermore, while most studies focus on topsoil P, the vertical distribution of P fractions throughout the soil profile is crucial for understanding long-term P sequestration, mobility, and root foraging strategies^10^. The patterns of labile versus occluded P with depth can reveal insights into the intensity of biological cycling and geochemical stabilization processes specific to different forest communities.

To address these gaps, we utilized a unique experimental system consisting of monocultures and all possible two-species mixtures of *R. pseudoacacia*, *P. orientalis*, and *Q. variabilis*. Our objectives were to: (1) assess how soil P forms and availability vary not only between pure and mixed stands, but also, and more importantly, among the different mixed-species combinations (*RQ*,* RP*,* PQ*); (2) characterize the vertical distribution (0–30 cm) of soil P fractions across these stand types; and (3) relate these patterns to key soil physicochemical properties. We hypothesized that: (i) mixed stands would exhibit higher labile P pools than monocultures, but the magnitude of this enhancement would be contingent upon the specific species pairing, with the greatest increase expected in the *RQ* mixture due to the complementary N-fixation and ECM-driven organic P mobilization strategies; and (ii) the proportion of occluded P (OP) in the total P pool would increase with soil depth across all stands, indicating a shift towards stabilized P forms in the subsoil.

## Materials and methods

### Study area

The study was conducted at the Henan Xiaolangdi Forest Ecosystem National Observation and Research Station, located along the Yellow River in Henan Province, China (35°01′ N, 112°28′ E)^11^. The region has a warm temperate continental monsoon climate, with a mean annual temperature of 12.4–14.3 °C and mean annual precipitation of 641.7 mm. The average annual evaporation is 1611.2 mm, and the annual sunshine duration is approximately 2367.7 h, yielding a sunshine rate of 54%. The frost-free period averages 213.2 days per year^12^. Dominant vegetation includes broadleaf forest, coniferous forest, scrub, and grasslands. The main soil types are derived from limestone weathering parent material and are classified as brown loamy soils. The characteristics of the six forest types are summarized in Table 1.

**Table 1:** Characteristics of tree stands in plots.

Factors	*R*	Q	*P*	RQ	RP	PQ
Forest age/ a	45	43	42	41	43	40
Slope aspect	South	South	North	South	South	North
Gradient/ ^o^	20	23	22	21	20	22
Mean height/ m	9.66 ± 0.57	8.75 ± 0.25	9.86 ± 0.16	10.95 ± 0.40	8.80 ± 0.31	9.86 ± 0.46
Mean DBH/ cm	11.35 ± 0.67	10.78 ± 0.30	10.64 ± 0.52	9.73 ± 0.56	10.80 ± 0.46	11.73 ± 0.26
Stand density/(plant/hm^2^)	1800 ± 100	1900 ± 100	1900 ± 100	1500 ± 100	1650 ± 50	1600 ± 50
Canopy density	0.82 ± 0.02	0.86 ± 0.06	0.90 ± 0.04	0.72 ± 0.04	0.80 ± 0.02	0.76 ± 0.03
Soil thickness/ cm	61	32	28	58	56	30

Note: *R*,* Robinia pseudoacacia* pure forest, *Q*, *Quercus variabilis* pure forest, *P*, *Platycladus orientalis* pure forest, *RQ*, *Robinia pseudoacacia-Quercus variabilis* mixed forest, *RP*, *Robinia pseudoacacacia- Platycladus orientalis* mixed forest, *PQ*, *Platycladus orientalis-Quercus variabilis* mixed forest. Data are presented as the mean ± standard deviation, the same as below.

### Soil sample collection and processing

Soil sampling was conducted in June 2022. Three 20 m × 20 m plots were established for each of the six forest types. After removing the litter layer, mineral soil samples were randomly collected at depths of 0–10 cm, 10–20 cm, and 20–30 cm using a stainless steel corer (5 cm inner diameter). To minimize spatial autocorrelation, a minimum distance of 40 m was maintained between any two adjacent plots.

From each plot, 16 sampling points were randomly selected. The soil cores from each depth were combined to form one composite sample per plot. Visible debris such as foliage, fine roots, and stones were carefully removed. The samples were air-dried in a cool, ventilated area, sieved through a 2 mm mesh, and divided into two portions. One portion was air-dried for the analysis of soil organic carbon (SOC), total organic carbon (TOC), total nitrogen (TN), total potassium (TK), available potassium (AK), and P forms. The other portion was stored at 4 °C for the analysis of nitrate nitrogen (NO₃⁻–N) and ammonium nitrogen (NH₄⁺–N).

### Analysis of soil physicochemical properties

Soil organic matter (OM) was determined using the potassium dichromate titration method^13^. TOC was measured by the external heating method with potassium dichromate^14^. TN was analyzed by the semi-micro Kjeldahl method. TK was determined by sodium hydroxide fusion–flame photometry, and AK was extracted with 1 mol/L ammonium acetate and measured by flame photometry. NO₃⁻–N was quantified using the phenol-sulfonic acid colorimetric method, and NH₄⁺–N was analyzed with 1.2 mol/L KCl extraction followed by the indophenol blue colorimetric method.

### Soil phosphorus fractionation

Soil P was fractionated using a modified Hedley sequential extraction procedure^15^. Briefly, 0.5 g of air-dried soil was sequentially extracted with: (1) 30 mL of 0.5 M NaHCO₃ (pH 8.5) for 16 h (Readily Reactive P, RRP); (2) 30 mL of 0.1 M NaOH for 16 h (Moderately Active P, MAP); and (3) 30 mL of 1 M HCl for 16 h after pre-ignition of the soil residue at 550 °C for 1 h (Occluded P, OP). After each extraction, the suspension was centrifuged at 4000 rpm for 10 min, and the supernatant was filtered through a 0.45-µm membrane. Inorganic P (Pi) in the NaHCO₃ and NaOH extracts was determined by the molybdenum-blue method. Total P (Pt) in the NaOH extract (after persulfate digestion) and the HCl extract represented organic+inorganic P and total OP, respectively. Organic P (Po) in the NaOH fraction was calculated as the difference between Pt and Pi. Available P (AP) was determined separately by the Olsen method.

### Statistical analysis

Statistical analyses were performed using SPSS 16.0 (IBM, USA). Given the hierarchical sampling design (three plots per forest type, with composite samples from three depths. The sample size for each forest type at each depth was *n* = 3.), data for each soil depth layer (0–10, 10–20, 20–30 cm) were analyzed separately to avoid pseudo-replication. Differences in soil P fractions and properties among the six forest types within each depth were assessed using one-way analysis of variance (ANOVA), followed by Tukey’s honestly significant difference (HSD) post-hoc test for multiple comparisons when ANOVA indicated significance (*P* < 0.05). Pearson correlation coefficients were calculated to examine relationships between P forms and soil physicochemical properties pooled across all samples and depths. While a linear mixed-effects model would be ideal to fully account for the nested structure, our separate ANOVA per depth provides a conservative and clear assessment of treatment effects at each soil layer.

## Results

### Characteristics of soil inorganic and organic phosphorus

**Fig. 1:**
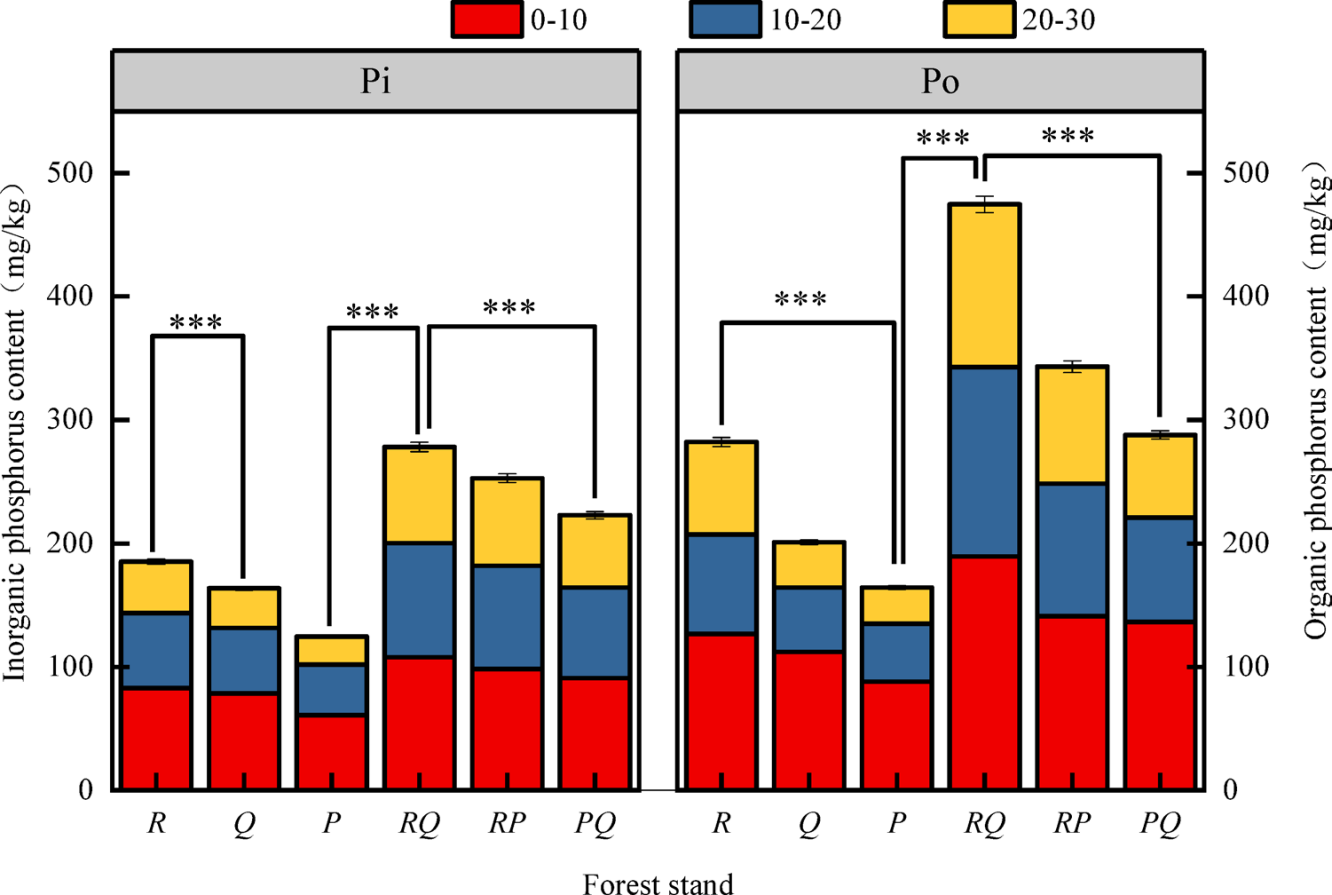
Characteristics of total inorganic phosphorus and total organic phosphorus in soils. Values are mean ± SE (*n* = 3 plots). Different lowercase letters above bars indicate significant differences among forest types within the same soil depth (*P* < 0.05, one-way ANOVA). Abbreviations: Pi, inorganic phosphorus; Po, organic phosphorus; R, *Robinia pseudoacacia*; Q, *Quercus variabilis*; P, *Platycladus orientalis*; *RQ*,* RP*,* PQ*, mixed forests.

Organic P was the dominant form of soil P across all six forest types (Fig. 1). The trends for both inorganic and organic P were consistent, showing a decrease in content with increasing soil depth. Among the 10–20 cm layer, the *RQ* forest exhibited the highest levels of inorganic and organic P, at 107.77 mg·kg⁻¹ and 189.56 mg·kg⁻¹, respectively. In contrast, the *P* forest had the lowest P content in the same layer, with values of 22.63 mg·kg⁻¹ for inorganic P and 29.34 mg·kg⁻¹ for organic P. In the 0–10 cm and 20–30 cm layers, the *R* forest showed the highest P concentrations, while the *PQ* forest had the lowest. ANOVA revealed that the total inorganic and organic P in the *RQ* forest differed significantly (*P* < 0.05) from the other five forest types across soil layers.

### Total phosphorus content

**Fig. 2:**
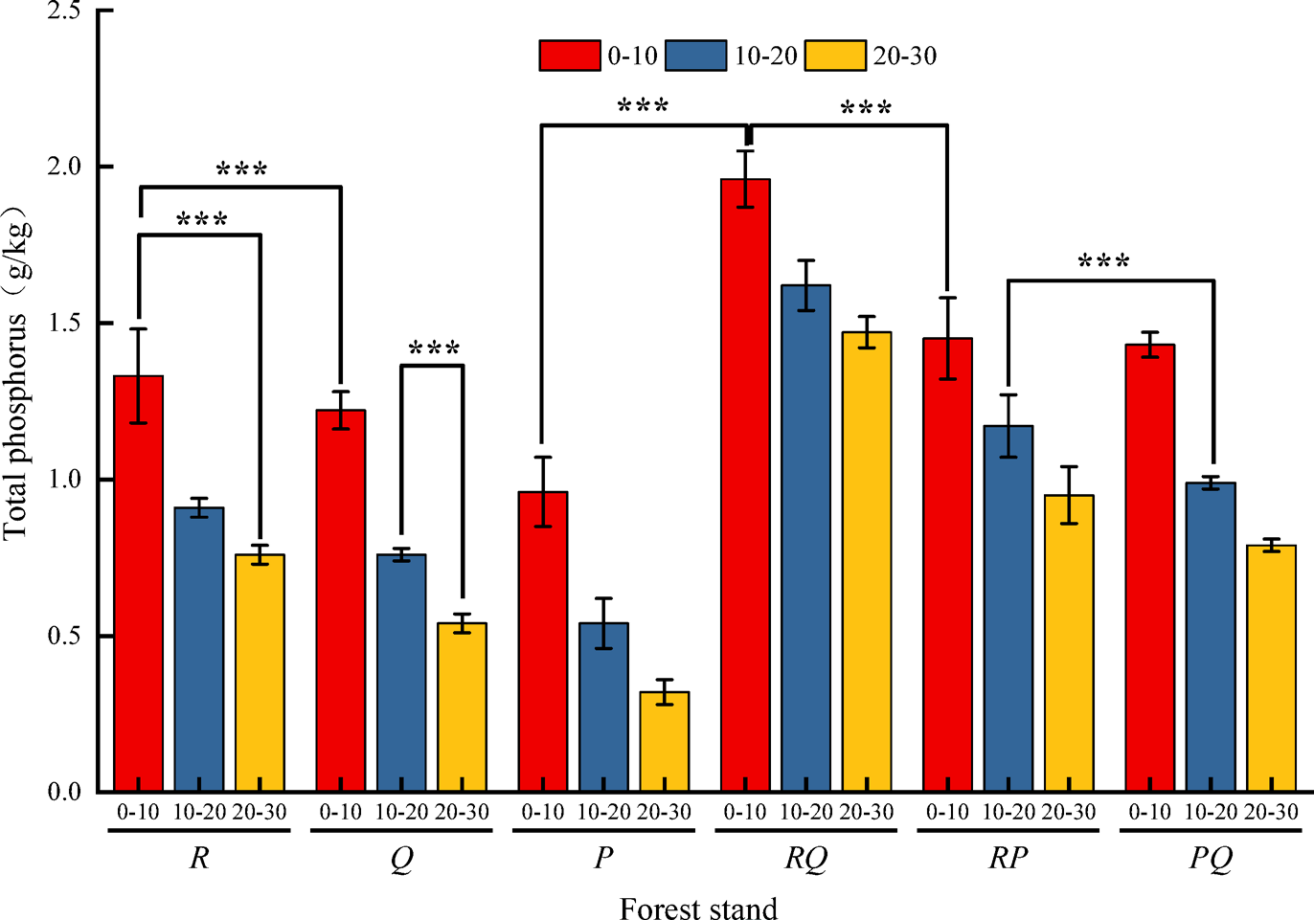
Characteristics of total phosphorus in soil. Values are mean ± SE (*n* = 3 plots). Different lowercase letters above bars indicate significant differences among forest types within the same soil depth (*P* < 0.05, one-way ANOVA). Abbreviations as in Fig. 1.

Total P content across the six forest types ranged from 0.32 to 1.96 g·kg⁻¹ (Fig. 2). The variation characteristics of total P were consistent with those of inorganic and organic P, decreasing with soil depth. Mixed forests generally had higher total P than pure forests. In the 0–10 cm layer, the *RQ* forest had the highest total P (1.96 g·kg⁻¹), while the *P* forest had the lowest (0.96 g·kg⁻¹). This pattern persisted in deeper layers. The total P in the *RQ* forest differed significantly (*P* < 0.05) from that in the other forests across different soil layers.

### Soil phosphorus fraction content

**Fig. 3:**
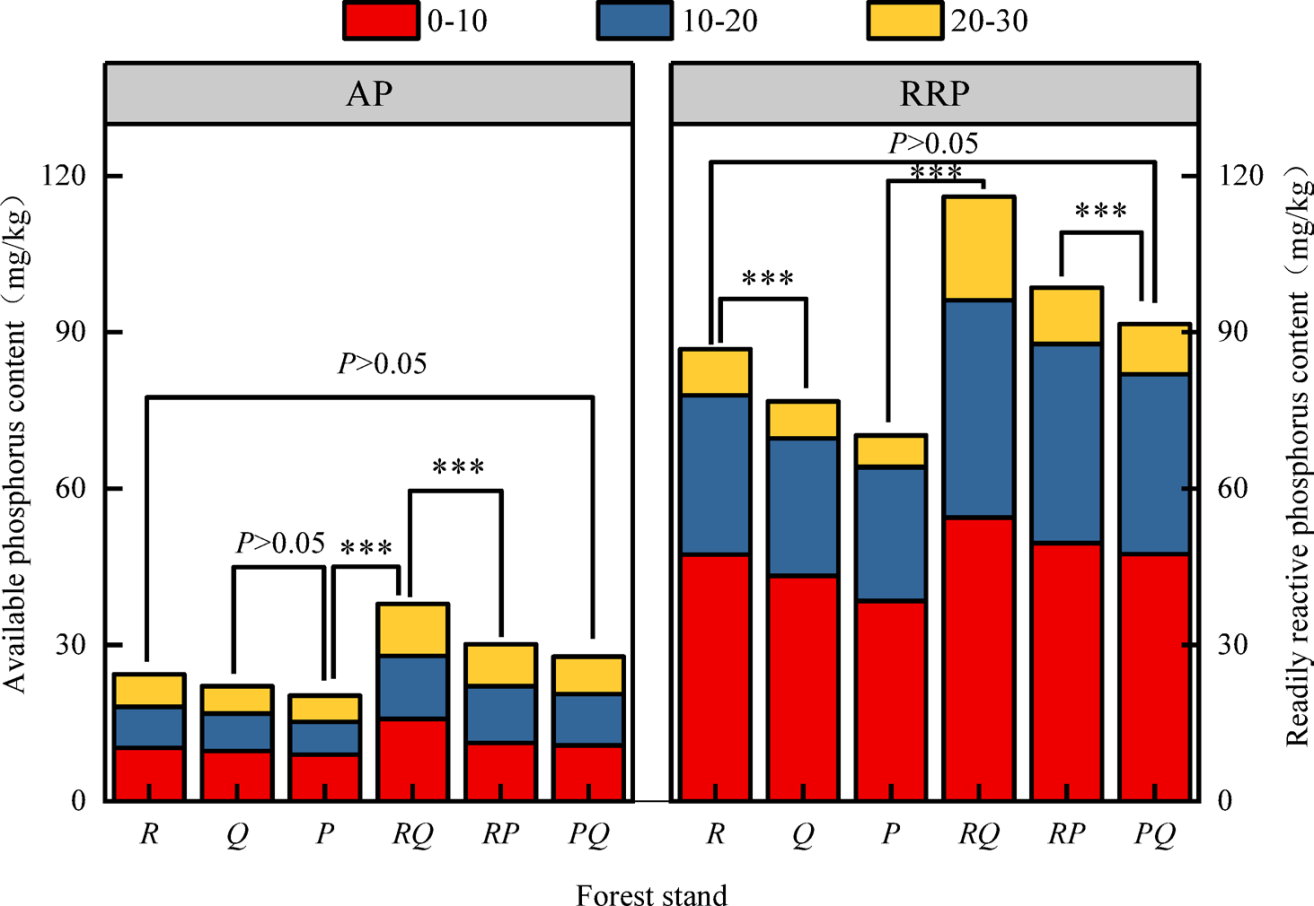
Characteristics of available phosphorus and readily reactive phosphorus in soil. Values are mean ± SE (*n* = 3 plots). Different lowercase letters above bars indicate significant differences among forest types within the same soil depth (*P* < 0.05, one-way ANOVA). Abbreviations: AP, available phosphorus; RRP, readily reactive phosphorus; other abbreviations as in Fig. 1.

The content of available P (AP) was significantly lower than that of readily reactive P (RRP) across all forest types (Fig. 3). AP ranged from 5.25 to 15.77 mg·kg⁻¹, while RRP ranged from 6.08 to 54.49 mg·kg⁻¹. The *RQ* forest had the highest AP values, and the *P* forest the lowest. No significant differences (*P* > 0.05) were found between some forest types in the 0–10 cm layer, but significant differences (*P* < 0.05) were observed in deeper layers. The AP content decreased significantly with depth (*P* < 0.05).

**Fig. 4:**
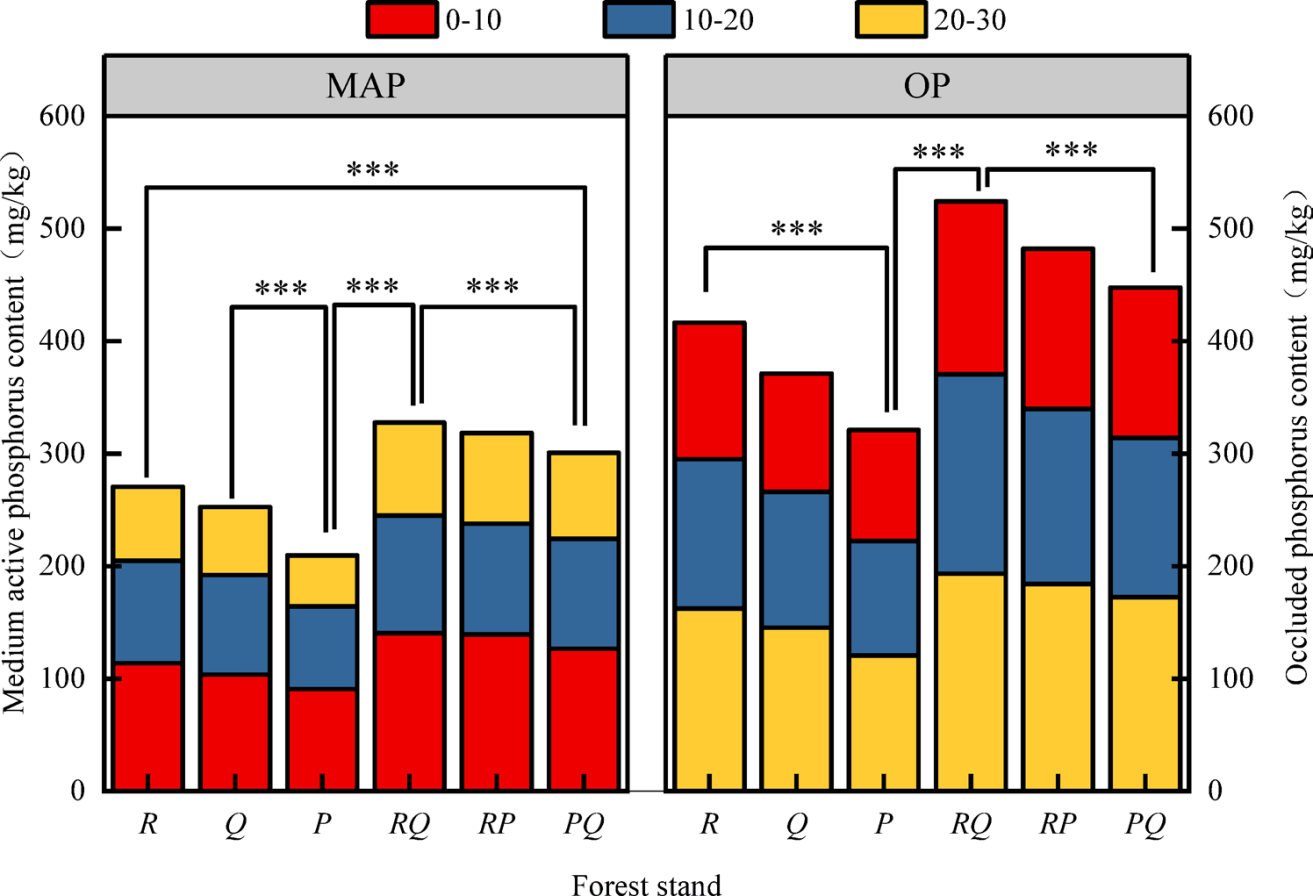
Characteristics of medium active phosphorus and occluded phosphorus in soils. Values are mean ± SE (*n* = 3 plots). Different lowercase letters above bars indicate significant differences among forest types within the same soil depth (*P* < 0.05, one-way ANOVA). Abbreviations: MAP, moderately active phosphorus; OP, occluded phosphorus; other abbreviations as in Fig. 1.

Moderately active P (MAP) and occluded P (OP) were the dominant fractions (Fig. 4). MAP ranged from 45.39 to 140.47 mg·kg⁻¹, and OP from 98.57 to 193.23 mg·kg⁻¹. The *RQ* forest had the highest MAP and OP contents across all layers. Mixed forests (*RQ*,* RP*,* PQ*) significantly increased MAP content compared to the P forest (*P* < 0.05). The overall order of P fractions was OP > MAP > RRP ≈ AP.

### Relationship between soil phosphorus and physicochemical properties

**Fig. 5:**
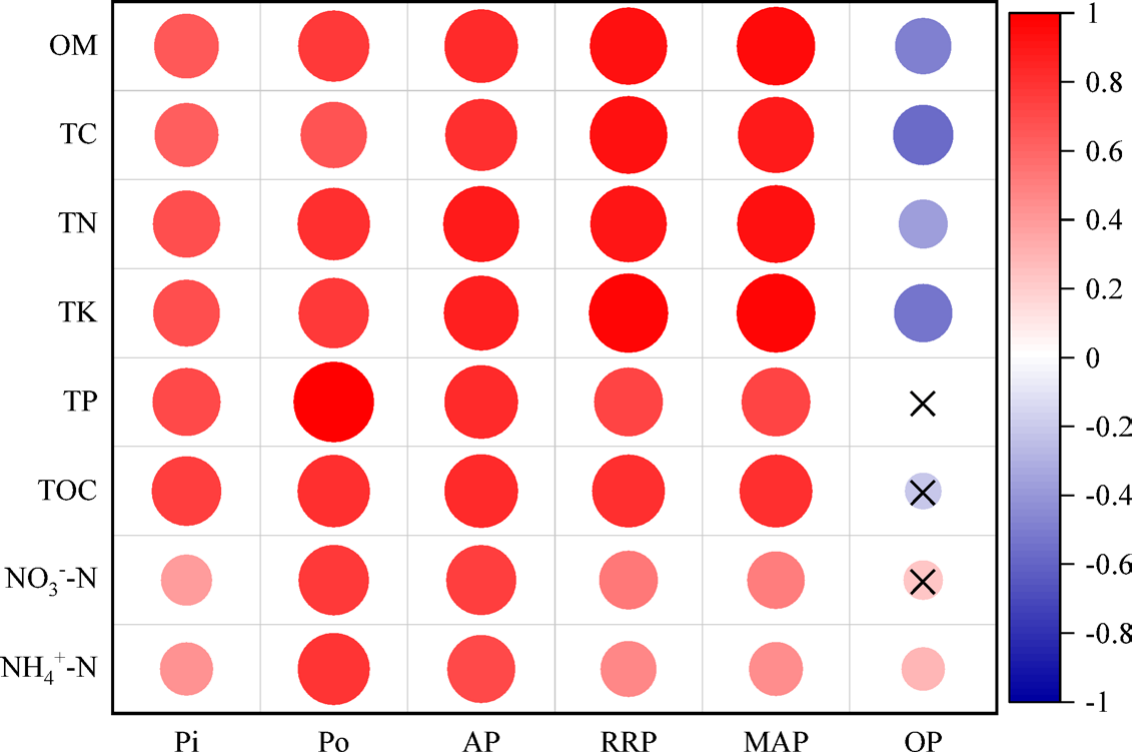
Correlation analysis between soil phosphorus forms and physicochemical properties. The heatmap displays Pearson correlation coefficients. Asterisks denote significance levels: *P < 0.05, **P < 0.01. Abbreviations: Pi, inorganic P; Po, organic P; AP, available P; RRP, readily reactive P; MAP, moderately active P; OP, occluded P; OM, organic matter; TC, total carbon; TN, total nitrogen; TK, total potassium; TP, total phosphorus; TOC, total organic carbon; NO₃⁻-N, nitrate nitrogen; NH₄⁺-N, ammonium nitrogen; AK, available potassium.

Inorganic P was significantly positively correlated (*P* < 0.01) with all soil physicochemical indicators (Fig. 5), with the highest correlation coefficients for TP (0.715) and TC (0.745). Organic P (Po) and AP also showed significant positive correlations with all indicators (*P* < 0.01). Po was most strongly correlated with TP, while AP correlated most strongly with TN. RRP and MAP were most strongly correlated with TK and OM, and weakly with NO₃⁻–N and NH₄⁺–N. In contrast, OP was significantly negatively correlated (*P* < 0.01) with other P forms and physicochemical properties, with the strongest correlations for TC and TK.

## Discussion

Our detailed analysis of soil P fractions across six plantation types reveals that the benefits of mixed-species planting on soil P status are not uniform, but critically depend on the identity of the constituent species. Furthermore, the vertical partitioning of P fractions, particularly the enrichment of occluded P in deeper layers, provides new insights into long-term P stabilization in these ecosystems. Below, we interpret these findings by integrating the functional traits of the studied tree species.

### The identity of species combinations dictates the outcome on soil phosphorus

The superior performance of the *R. pseudoacacia*–*Q. variabilis* (RQ) mixture across multiple P metrics (total P, organic P, available P; Figs. 1, 2 and 3) strongly supports the hypothesis that functional complementarity drives synergistic effects^16^. This combination pairs a nitrogen-fixing legume (*Robinia*) with an ECM-associated oak (*Quercus*). We propose a mechanistic interplay: *Robinia*, through biological N₂ fixation, likely elevates soil N availability (supported by higher TN in *RQ* stands, Fig. 5), which can stimulate microbial biomass and activity, including the production of phosphatases that mineralize organic P^17,18^. Concurrently, *Q. variabilis*, with its ECM associations, is proficient at mining organic and mineral-bound P pools through extensive hyphal networks and exudation of weathering agents^9^. The N input from *R. pseudoacacia* may thus “prime” the microbial community, while the ECM fungi from *Q. variabilis* enhance the mobilization of P from less available forms, creating a positive feedback loop for P cycling. In contrast, the more modest or inconsistent effects observed in the *RP* and *PQ* mixtures underscore the importance of species identity. The *RP* mixture, while combining a N-fixer with an ECM conifer (*P. orientalis*), may not achieve the same synergy. This could be due to competition for similar inorganic P pools, or a potential mismatch in belowground carbon investment strategies, where the carbon cost of maintaining ECM symbionts in *P. orientalis* is not efficiently offset by the neighboring *Robinia*’s N input. The *PQ* mixture, comprising two ECM-associated species, may represent a case of functional redundancy in terms of primary P acquisition strategy, limiting the potential for niche differentiation and strong facilitative interactions^19^. This spectrum of responses—from pronounced synergy in *RQ* to weaker effects in *RP* and *PQ*—provides compelling field evidence that the outcome of tree species mixing on soil nutrients is contingent on the specific functional traits brought together^8^.

### Vertical stratification of phosphorus fractions: implications for sequestration and supply

A consistent pattern across all stands was the decrease in total and labile P concentrations with soil depth (Figs. 1, 2, 3 and 4), reinforcing the surface layer as the hotspot of biological P cycling^10^. The most striking vertical finding was the peak of occluded P (OP, 193.23 mg·kg⁻¹) in the 20–30 cm layer of the *RQ* forest (Fig. 4). OP represents P bound within stable mineral matrices or strongly adsorbed to surfaces, and its accumulation in the subsurface suggests processes of P translocation and stabilization over time. We hypothesize two non-exclusive mechanisms linked to the *RQ* combination: (i) Enhanced deep root activity and turnover: The potentially complementary root systems of deep-rooting *Robinia* and *Quercus* may lead to greater root-derived organic matter input at depth. Subsequent decomposition and release of P could be followed by re-adsorption or precipitation into stable forms in the subsoil^20^. (ii) Leaching and re-stabilization: Dissolved organic or inorganic P complexes generated from the highly active, organic-rich topsoil of the *RQ* stand may leach downward and be immobilized upon encountering different mineralogy or pH conditions in the subsurface. The strong negative correlation between OP and labile P fractions as well as OM and TC (Fig. 5) supports its role as a long-term sink. This vertical partitioning highlights that assessments of P cycling must consider the entire soil profile, as subsoil processes may significantly influence long-term P sequestration and the resilience of the P supply.

### Links to soil properties and biological activation

The significant positive correlations between labile P fractions (AP, RRP, MAP) and soil organic matter (OM), total carbon (TC), and total nitrogen (TN) (Fig. 5) underscore the foundational role of soil organic matter in sustaining P bioavailability. Organic matter fuels the microbial engine that drives P mineralization and produces ligands that compete with phosphate for sorption sites^21,22^. The elevated levels of these fertility indicators in the *RQ* mixture likely created a favorable environment for microbial-mediated P transformations. This biological activation appears to be a key mechanism behind the observed P availability benefits in the most complementary mixture, moving beyond a simple dilution or physical mixing effect.

### Limitations and future perspectives

While our findings highlight the promise of specific species mixtures like *RQ*, we acknowledge limitations inherent to our field-based study. Pre-existing site variations in slope aspect (south-facing for *R*, *Q*, *RQ*, *RP* vs. north-facing for *P*, *PQ*) and soil thickness (Table 1) may influence microclimate and water dynamics, potentially confounding the vegetation effects^23^. Although we employed a replicated sampling design to capture representative conditions, causal attribution solely to species mixing requires caution. Our conclusions should therefore be interpreted as revealing strong associations under the prevailing environmental context.

Future research should employ common garden experiments with controlled planting designs to isolate the effects of species identity and interaction. Integrating measurements of root biomass and distribution, mycorrhizal colonization rates, microbial community composition, and phosphatase activity would directly test the mechanistic pathways proposed here. Long-term monitoring is essential to determine if the observed patterns, especially the deep OP accumulation, are stable over decadal scales. Finally, practical studies on the establishment and management economics of these targeted mixed plantations are needed to translate ecological insights into actionable forestry strategies.

## Conclusion

This study provides a systematic comparison of soil P dynamics in six plantation types at the southern foot of the Taihang Mountains. The results indicate that mixed-species plantations, particularly the *R. pseudoacacia–Q. variabilis* mix, were associated with enhanced soil P storage and availability compared to monocultures. Organic P dominated the soil P pool, and all P forms decreased with soil depth, highlighting the critical role of the surface layer in nutrient cycling. The predominance of occluded P across all stands indicates a large stable P reservoir, the dynamics of which are closely tied to soil organic matter and total carbon.

From a practical perspective, we recommend that forest management in this region and similar temperate ecosystems should:

1. Prioritize mixed-species afforestation strategies to enhance soil fertility and ecosystem sustainability.
2. Implement surface soil conservation measures to protect the nutrient-rich topsoil from erosion.
3. Promote organic matter inputs to stimulate the biological cycling of phosphorus. These recommendations should be applied with consideration of local site conditions, including slope, soil depth, and historical land use.

Future research should focus on long-term monitoring of P transformations, with emphasis on microbial-mediated processes and the scalability of mixed-forest benefits, thereby informing more precise environmental assessment models.

## Data Availability

All data generated or analyzed during this study are included in this published article.
